# Prolonged respiratory failure responds to conventional therapy in isolated homocysteine remethylation defects

**DOI:** 10.1002/jmd2.12375

**Published:** 2023-06-16

**Authors:** Abigail Whitehouse, Preeya Rehsi, Louise Hartley, Stephanie Grunewald, Berna Seker Yilmaz, Kelly Pegoretti Baruteau, Ayhan Yaman, Suren Thavagnanam, Julien Baruteau

**Affiliations:** ^1^ Department of Paediatric Respiratory Medicine, Barts Health NHS Trust Royal London Hospital London UK; ^2^ Department of Metabolic Medicine Great Ormond Street Hospital for Children NHS Foundation Trust London UK; ^3^ Department of Paediatric Neurology Barts Health NHS Trust, Royal London Hospital London UK; ^4^ Great Ormond Street Institute of Child Health University College London London UK; ^5^ Department of Neuroradiology University College London Hospitals NHS Foundation Trust London UK; ^6^ Department of Paediatric Intensive Care Unit İstinye Üniversite Hastanesi Liv Hospital Istanbul Turkey

**Keywords:** CblG, cobalamin, homocysteine, inherited metabolic disease, isolated remethylation defect, MTHFR, respiratory failure, tracheostomy

## Abstract

Isolated remethylation defects are rare inherited diseases caused by a defective remethylation of homocysteine to methionine, preventing various essential methylation reactions to occur. Patients present with a systemic phenotype, which can especially affect the central and peripheral nervous systems leading to epileptic encephalopathy, developmental delay and peripheral neuropathy. Respiratory failure has been described in some cases, caused by both central and peripheral neurological involvement. In published cases, the genetic diagnosis and initiation of appropriate therapy were rapidly performed following respiratory failure and led to a rapid recovery of respiratory insufficiency within days. Here, we present two infantile‐onset cases of isolated remethylation defects, cobalamine (Cbl)G and methylenetetrahydrofolate reductase (MTHFR) deficiencies, which were diagnosed after several months of respiratory failure. Disease modifying therapy based on hydroxocobalamin and betaine was initiated and shows a progressive improvement and enabled weaning off respiratory support after 21 and 17 months in CblG and MTHFR patients respectively. We show that prolonged respiratory failure responds to conventional therapy in isolated remethylation defects, but can require a sustained period of time before observing a full response to therapy.

**Synopsis**

Respiratory failure is a well‐described symptom in isolated remethylation defects. Respiratory failure in isolated remethylation defects responds well to conventional therapy, even when the therapy is started with prolonged delay after the start of the respiratory symptoms.

## 1 INTRODUCTION

Isolated remethylation defects (IRDs) are rare inherited disorders caused by defective remethylation of homocysteine in methionine, which prevents the synthesis of S‐adenosylmethionine (SAM), the main substrate for the numerous and critical methylation reactions required in physiology.^1^ Patients with IRD will present with a multisystemic phenotype, affecting the central and peripheral nervous systems, the bone marrow and the kidneys. Clinical presentations vary considerably according to the age of onset and the genetic defect. Infantile‐onset diseases present with a more severe phenotype.^2^ CblE and CblG defects typically show megaloblastic anaemia, hypotonia and neurocognitive impairment, with occasionally seizures, loss of consciousness and visual impairment. CblE and CblG are encoded by the *MTRR* and the *MTR* genes respectively, synthesising the methionine synthase enzyme and the methionine synthase reductase, an accessory protein required to maintain methionine synthase bound to cobalamin in its active form.^3^ MTHFR deficiency usually presents in early childhood with feeding difficulties, hypotonia, cognitive impairment, apnoea but no haematological signs.^4^ Juvenile and adult presentations include neuropsychiatric symptoms with occasionally megaloblastic anaemia.^2^ This clinical presentation is as well observed in CblD‐HC.^5^ In all four disorders, hyperhomocysteinaemia, hypomethioninaemia with absence of methylmalonic aciduria are typical biochemical presentations.^1^

Respiratory insufficiency is a well‐recognised complication of these disorders, caused by involvement of both central and peripheral nervous systems.^6^ This symptom responds well to disease‐modifying therapy when the treatment is initiated soon after the start of respiratory symptoms. There are no previous case reports on the respiratory prognosis of IRD that occur with already prolonged established respiratory failure. Here, we present two patients with infantile‐onset IRD, case 1 with CblG deficiency and case 2 with MTHFR deficiency, who were diagnosed and commenced on disease‐modifying therapy after several months of established respiratory insufficiency. Respiratory failure responded well to therapy and the respiratory support was weaned off after 21 and 17 months respectively, of therapy based on hydroxocobalamin and betaine. We show that prolonged respiratory failure is a curable symptom in IRD, although a sustained period of time is necessary before achieving a full response to therapy.

## 2 PATIENTS AND METHODS

We reviewed the long‐term evolution of two patients with IRD, who were genetically diagnosed after a prolonged period of respiratory failure with clinical details summarised in Table 1. Both families provided written consent for publication.

**TABLE 1:** Main diagnostic and therapeutic features for both patients.

	Patient 1	Patient 2
Diagnosis	cblG deficiency	MTHFR deficiency
Genotype	*MTR* gene. Compound heterozygous mutations c.3518C > T (p.Pro1173Leu)/c.3600del (p.Ile1201Leufs)	*MTHFR* gene. Pathogenic intronic mutation NM_005957.4, c.1753‐18G > A
Age of onset of symptoms (months)	6	1
Age at start of treatment (months)	11.5	20
Metabolic management	Hydroxycobalamin 1 mg IM (increased to 4 mg/injection SC, 4 days a week) Betaine 250 mg/kg/day oral Methionine 100 mg TDS oral Calcium folinate 15 mg/day oral	Betaine 450 mg/kg/day (3 divided doses) oral Calcium folinate 15 mg/day oral
Respiratory management	Tracheostomy 11–32 m Decannulation 32 m	Tracheostomy 7–24 m Decannulation at 24 m
Mode of ventilation via tracheostomy—iPAP/PEEP	P‐SIMV—+12/+6 cmH2O	P‐SIMV—+20/+5 cmH2O

Abbreviations: IM, intramuscular; iPAP, inspiratory positive airway pressure; PEEP, positive end expiratory pressure; P‐SIMV, pressure synchronised intermittent mandatory ventilation; SC, subcutaneous; SIMV, synchronised intermittent mandatory ventilation; TDS, three times a day.

## 3 PATIENT 1

### 3.1 Clinical presentation

This patient is the second child of unrelated parents. She was born at term after an uneventful pregnancy and presented with failure to thrive, developing anorexia for milk and solid food from the age of 5 months. At 6 months of age, she was treated as an outpatient for a lower respiratory tract infection with amoxicillin‐clavulanic acid. Subsequently over some weeks she started to show increasing respiratory distress, with recession and head bobbing. In parallel, and although her neurodevelopment had been normal until 6 months of age, she showed evidence of neuro‐regression with progressive generalised hypotonia, reduced mobility, hypomimic face, reduced smiling, difficulty to grab objects and move her limbs. She became much sleepier. Initial investigations at 10 months of age were reported as normal (plasma amino acids including methionine at 19 μmol/L (*N* 10–60), ammonia, creatine kinase, very long chain fatty acids, lactate, electroencephalogram). An elevated homocysteine level was reported at 107 μmol/L (*N* < 15) but not investigated further. Brain MRI was reported to be normal. At 11 months of age, the child was admitted to her local general hospital to start nutritional support via nasogastric tube. During this admission, she desaturated to 70% oxygen resulting in intubation and transfer to a tertiary paediatric intensive care unit.

### 3.2 Diagnosis and therapy

**FIGURE 1:**
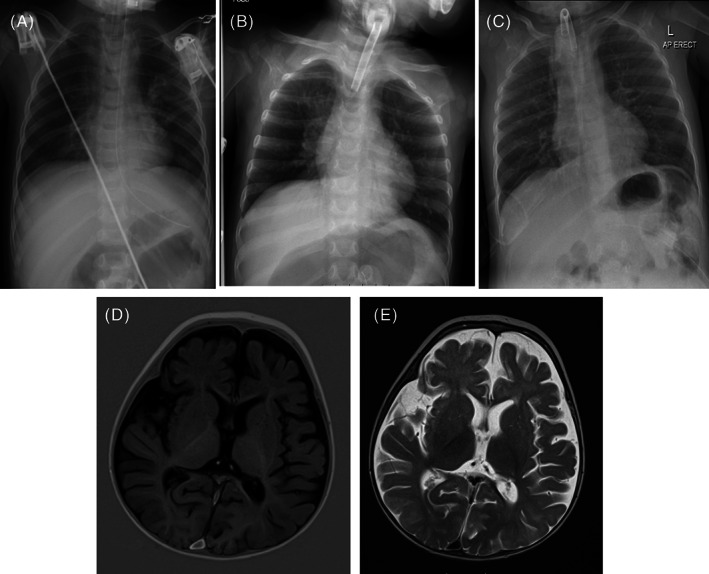
Imaging from Case 1. Chest X‐rays before (A), 3 months (B) and 11 months (C) after initiation of ventilatory support. (A) Both diaphragms are flattened and at equal levels suggesting abnormal dynamics of the diaphragms. (B) The right hemi‐diaphragm is ascended with tracheostomy in situ. (C) The diaphragms look in physiological position with tracheostomy in situ. Brain MRI T1 (D) and T2 (E) sequences showing cerebral atrophy and delayed myelination predominant in the frontal lobes at 11 months of age.

Chest X‐ray showed bilaterally flattened diaphragms with both diaphragms at equal levels (Figure 1A). A diaphragmatic ultrasound identified reduced diaphragmatic movement bilaterally and reduced diaphragmatic excursion on M‐mode imaging suggesting diaphragmatic paralysis. There was no evidence of paradoxical movement. Echocardiography was normal. Electromyogram studies suggested sensory neuropathy with sensory responses unobtainable in lower limbs, potentially impacted by movement artefact, but normal motor studies. A repeated brain MRI showed mild generalised brain atrophy and delay in myelination (Figure 1D,E). Cerebrospinal fluid (CSF) analysis showed normal biochemistry and cellularity, including CSF lactate, amino acids and oligoclonal bands being unremarkable. A rapid trio exome subsequently identified compound heterozygous mutation of *MTR* gene c.3518C > T (p.Pro1173Leu)/c.3600del (p.Ile1201Leufs). By that time, the child had profound hypotonia with no deep tendon reflexes, muscular strength scored 1/5 in proximal limbs, 2–3/5 in distality, following with her eyes but not being able to move her head independently. She was entirely fed by NG tube and required ventilation support via nasotracheal tube.

Following diagnosis, she was started on hydroxocobalamin 1 mg/day intramuscularly, betaine citrate 250 mg/kg/day and calcium folinate 15 mg/day orally. Methionine supplementation was started at 200 mg/day orally for 3 weeks then increased to 300 mg/day. Methionine levels normalised rapidly from 9 μM pretreatment to 40–68 μmol/L (*N* 10–60). Homocysteine levels fell from 107 and 137 μmol/L to 20 μmol/L (*N* < 15). After 12 months of therapy, she was started on subcutaneous injection of hydroxocobalamin increased progressively from 1 to 4 mg daily, and spaced out from 7 to 4 injections per week enabling stable methionine and homocysteine levels.

### 3.3 Respiratory function

After 3 weeks of treatment, no improvement was seen on diaphragmatic ultrasound. After three failed extubation attempts, a tracheostomy was inserted and percutaneous gastrostomy was placed, 21 days after having started the disease‐modifying therapy. The child was on inspiratory positive airway pressure (iPAP) and positive end expiratory pressure (PEEP) of +12 and +6 cmH2O respectively. At 3 months post‐initiation of therapy (Figure 1B), further diaphragm ultrasound showed vastly improved movement of both diaphragms. She was noted to start having occasional hiccups. Her 24‐h BiPAP requirement via tracheostomy was gradually weaned to increasing durations on CPAP with PEEP initially at +9 cmH2O whilst the chest X‐ray showed a physiological aspect of the diaphragms (Figure 1C). The patient showed excellent progress after discharge home and following two subsequent planned admissions, alongside sleep studies, her ventilation was weaned. She was then successfully decannulated at 32 months of age with no ongoing need for ventilatory support. Together with her improved muscle strength, her cough reflex improved and she did not suffer from recurrent chest infections.

### 3.4 Neurodevelopment under treatment

After 3 weeks of therapy, the child was moving all four limbs with increased muscular strength to score 3–4/5. She was starting to roll over, became more interactive with her environment, wanting to grab objects, smiling frequently with various facial expressions. After 20 weeks of therapy, she was able to sit, interacting very well, waving, clapping, speaking single words and tolerating some oral feeds. At 2 years of age, she was walking independently and speaking 10 words. At 4 years of age, she has caught up in regards of her motor development and communication. She is performing the same outdoor activities as any child of her age like swimming, cycling or jumping in a trampoline. She showed a mild speech delay in the 12 months following her decannulation, which could be attributed to her prolonged tracheostomy. This has now resolved fully resolved and she is speaking complex sentences with appropriate vocabulary for her age.

Growth: At birth, her weight measured on the 96th centile, length on the 86th centile and head circumference on 80th centile. At initiation of therapy, her weight, height and head circumference were on the 3rd, 75th and 10th centiles respectively. At most recent follow up aged 4 years, her recent weight, height and head circumference were on 27th, 35th and 5th centiles respectively.

## 4 PATIENT 2

### 4.1 Clinical presentation

This patient is the first child of non‐consanguineous parents, who originate from the same village in Turkey. Her mother suffers from Familial Mediterranean Fever and had been treated for Hodgkin's lymphoma. The patient was born at 41 + 5 weeks gestation by spontaneous vaginal delivery in the UK following an uneventful pregnancy. She weighed 3.33 kg at birth. She was admitted for 5 days to the neonatal unit for neonatal respiratory distress and suspicion of infection, requiring oxygen supplementation and antibiotics, which rapidly resolved.

**FIGURE 2:**
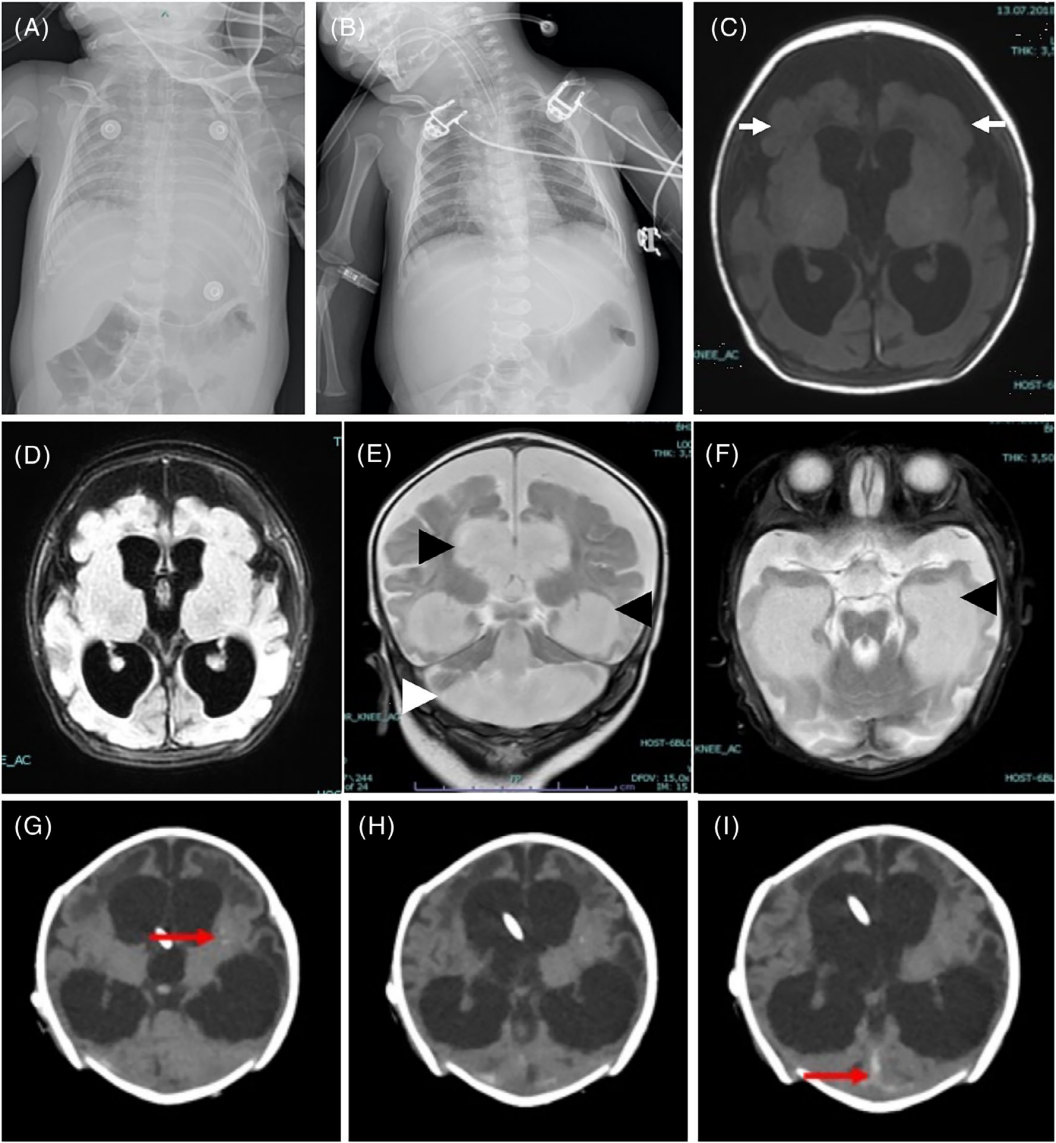
Imaging from Case 2. Chest X‐rays before (A) and after (B) initiation of ventilatory support. (A) Bilateral atelectasis with hypoventilation. (B) Ventilatory support with tracheostomy in situ, bilaterally ascended diaphragms suggesting diaphragmatic dysfunction. Brain MRI T1 (C), FLAIR (D) and T2 (E, F) sequences showing generalised paucity of cortical folding (C, white arrows), hydrocephalus with volume loss of cerebral parenchyma (E, F, black arrows), cerebellar vermis hypoplasia and mega cisterna magna (E, white arrowhead) at 3 months of age. Brain computed tomography (G–I) showing basal ganglia calcifications (G, read arrow) and venous sinus thrombosis (I, red arrow) with derivation of the hydrocephalus.

At 1 month of age, the infant travelled to visit family in Turkey. Concomitantly she developed epileptic spasms, which precipitated hospital admission. An electroencephalogram performed revealed an epileptic encephalopathy and she was commenced on levetiracetam, vigabatrin, phenobarbitone and pyridoxine. She progressively developed poor feeding, signs of neuro‐regression, became lethargic and hypotonic with reduced interaction and eye contact. A video fluoroscopy performed at 2 months of age was complicated by aspiration pneumonia. She was intubated and admitted to the paediatric intensive care unit (Figure 2A,B). Extubation attempts failed and weaning of ventilation was unsuccessful. A brain computed tomography scan revealed a communicating tetraventricular hydrocephalus and a right venous sinus thrombosis, together with basal ganglia calcifications that were interpreted as secondary to infection. A brain MRI revealed progression of the hydrocephalus at 3 months of age, which led to the insertion of a ventriculo‐peritoneal shunt (Figure 2C–I). She developed CMV infection for which she received ganciclovir. The child was diagnosed and managed in Turkey initially before returning to the United Kingdom at 3.5 years of age.

### 4.2 Diagnosis and treatment

At 20 months of age, whole genome sequencing identified MTHFR deficiency with homozygous pathogenic intronic mutation c.1753‐18G > A in the *MTHFR* gene. Plasma biomarkers showed elevated homocysteine at 297 μmol/L and low methionine at 7 μmol/L. She was started on betaine 450 mg/kg/day and calcium folinate 15 mg daily. This enabled a decrease of total homocysteine to 70–80 μmol/L and normalised plasma methionine levels. However CSF 5‐methyltetrohydrofolate levels remain undetectable (<10 nmol/L, 52–178 nmol/L) at 4 years of age.

### 4.3 Respiratory function

Patient was intubated and ventilated from 2 months of age. A tracheostomy was placed at 7 months of age. The patient sustained a total of 22 months of invasive ventilation. Four months after initiation of betaine and calcium folinate therapy, her respiratory function improved and she was weaned from ventilatory support at 24 months of age. At 4 years of age, stimulation of the phrenic nerve revealed reproducible responses suggesting at least partial innervation of the diaphragm bilaterally.

### 4.4 Neurodevelopment under treatment

Following seizures at 1 month of age well‐controlled by anti‐seizure medication, the patient remained seizure‐free until the age of 2 years. Focal complex seizures were well controlled by rescue benzodiazepine as well as regular levetiracetam, and she was weaned off phenobarbitone. She showed severe global developmental delay with slow developmental progress. She acquired unsupported sitting at the age of 3.5 years. At the age of 3 years and 8 months, she could roll over but could not crawl or stand. She suffered from hyperkinetic movement of all four limbs. Her visual assessment showed large esotropia with roving eye movements, normal retinal function but flash visual evoked potentials were suggestive of rudimentary vision. Currently, she communicates by smiling to familiar faces, some babbling but has no speech and limited understanding.

### 4.5 Growth parameters

At birth, her weight measured on the 42nd centile, length on the 95th centile and head circumference on 19th centile. At time of initiation of therapy, aged 20 months, her weight, height and head circumference measured 75th, 3rd and 85th centiles respectively. At most recent follow up aged 4 years her weight falls on the 9th centile, height on the 25th centile and head circumference on <0.4th centile.

## 5 DISCUSSION

Both cases present the typical infantile‐onset of two different IRDs, CblG and MTHFR deficiencies. In case 1 with CblG deficiency, an insidious onset of symptoms was observed with failure to thrive, food refusal followed by neuro‐regression and respiratory insufficiency. In case 2 with MTHFR deficiency, a subacute onset of neurological symptoms with epilepsy, encephalopathy and hydrocephalus was reported in infancy complicated by respiratory failure. Both cases showed prolonged respiratory failure requiring long‐term invasive ventilation via tracheostomy. Following genetic diagnosis, a disease modifying therapy based on hydroxycobalamin and betaine enabled a progressive response and the weaning of artificial ventilation.

Respiratory failure is a well‐documented complication in infantile‐ and juvenile‐onset in both isolated and combined remethylation defects.^2^ In the natural course of these disorders, respiratory symptoms are rarely the first symptom to be reported, but will secondarily occur and progress rapidly to become one of the main signs compromising survival. The pathophysiology remains poorly understood with suggested toxicity of homocysteine, methionine deficiency interfering with methylation capacity, and increased oxidative stress being suggested as contributing factors.^1^ The understanding of the respiratory failure is incomplete but is likely multifactorial encompassing both peripheral and central neurological involvement. Diaphragmatic paralysis has been implicated in several cases described in the literature as demonstrated in case 1. Broomfield et al. described three cases with both radiological and electrophysiological evidence of diaphragmatic paralysis.^6^ This was followed by rapid resolution of clinical symptoms and electrophysiology upon initiation of treatment in all patients. The pathophysiology of both central and peripheral neuropathy is likely secondary to deficiencies in CSF SAM, a key methyl donor for multiple methylation reactions. This can result in demyelination in the central and peripheral nervous systems.^7^ The diaphragmatic paralysis is likely to be secondary to peripheral neuropathy affecting both phrenic nerves. Ultrasound exam of the diaphragm enables diagnosis of diaphragmatic paralysis and assesses response to treatment.

The presented cases in our report showed full reversibility of respiratory failure despite prolonged periods of respiratory symptoms before initiation of therapy. Case 1 developed respiratory symptoms 5 months before initiation of therapy and was weaned off ventilatory support after 21 months of therapy and 22 months of artificial ventilation. Similarly, case 2 developed respiratory insufficiency 19 months before initiation of therapy and was weaned off ventilatory support after 4 months of therapy and 17 months of artificial ventilation. This differs from previous published cases of isolated or combined remethylation defects with respiratory failure where the recovery following initiation of therapy was shorter.^6^ For example, a juvenile‐onset CblG‐deficient patient aged 9 years old was weaned of ventilatory support within 5 days following initiation of therapy.^6^ Both infantile‐ and juvenile‐onset MTHFR‐deficient patients aged 4 months and 6 years respectively, did not require ventilatory support after 7 and 2 days following the start of treatment.^6^ This suggests a correlation between duration of respiratory symptoms and time taken to respond following initiation of disease modifying therapies. In addition, evidence of peripheral neuropathy was observed on nerve conduction studies, with involvement of phrenic, posterior tibial, median and ulnar nerves. Similarly in case 1, a sensory neuropathy of the lower limbs was suspected. An adult‐onset CblC‐deficient woman aged 33 years presented with neuropsychiatric symptoms followed by respiratory insufficiency requiring ventilation. The respiratory symptoms recovered rapidly after initiation of therapy.^8^ Another report of adult‐onset CblC deficiency with respiratory failure described weaning from the ventilator within 24 h following hydroxycobalamin supplementation.^9^ Our cases highlight that even late therapy allows a gradual but complete clinical response of respiratory symptoms. Betaine enables an increase CSF S‐adenosyl methionine concentrations, thereby highlighting the importance of this methyl donor in the pathophysiology of peripheral and central neuropathy in these disorders.^10^, ^11^

These two reported cases also highlight the different neurological pathophysiology and response to therapy of the two disorders described. The patient with CblG deficiency presents with normal neurology at the age of 4 years whilst the patient with MTHFR deficiency suffers from severe developmental delay and is non‐verbal at a similar age. The natural history of untreated early‐onset MTHFR deficient patients results in profound neurocognitive impairment, severe cerebral atrophy and potentially hydrocephalus.^12^ Early identification and treatment of these conditions has been shown to improve short term outcomes in patients.^1^, ^13^ In cases of pre‐symptomatic treatment, short term neurodevelopmental outcomes in MTHFR and CblG deficiency patients can be normal.^4^ Since methionine levels are normal to low, these patients are not picked up in newborn screening, which uses methionine measurements on blood spot samples for the screening of homocystinuria. Nevertheless, development of newborn screening methods for IRD should be considered to allow early diagnosis and treatment given short window of opportunity for positive outcomes.

In summary, we present two cases of infantile‐onset IRD with severe respiratory failure at diagnosis, requiring prolonged invasive ventilatory support, who demonstrated complete reversibility of respiratory symptoms on disease modifying therapy. This provides essential information for counselling patients and families at diagnosis regarding long‐term prognosis.

## FUNDING INFORMATION

Julien Baruteau is supported by funding from the United Kingdom Medical Research Council Clinician Scientist Fellowship MR/T008024/1 and NIHR Great Ormond Street Hospital Biomedical Research Centre. The views expressed are those of the author(s) and not necessarily those of the NHS, the NIHR or the Department of Health.

## CONFLICT OF INTEREST STATEMENT

The authors have no conflict of interest to declare.

## ETHICS STATEMENT

Written consent for publication was obtained from patients' parents.

## Data Availability

The authors do not have authorisation from the families to share personal data. Sharing anonymised data can be discussed upon request to Dr. Julien Baruteau.
